# Supplementary material for: Direct identification of clinically relevant neoepitopes presented on native human melanoma tissue by mass spectrometry
Source: Nat Commun. 2016 Nov 21;7:13404. doi: 10.1038/ncomms13404 (PMC5121339; doi:10.1038/ncomms13404)
Supplement: Supplementary Information — Supplementary Figures 1-24 and Supplementary Tables 1-3. [file ncomms13404-s1.pdf]

a

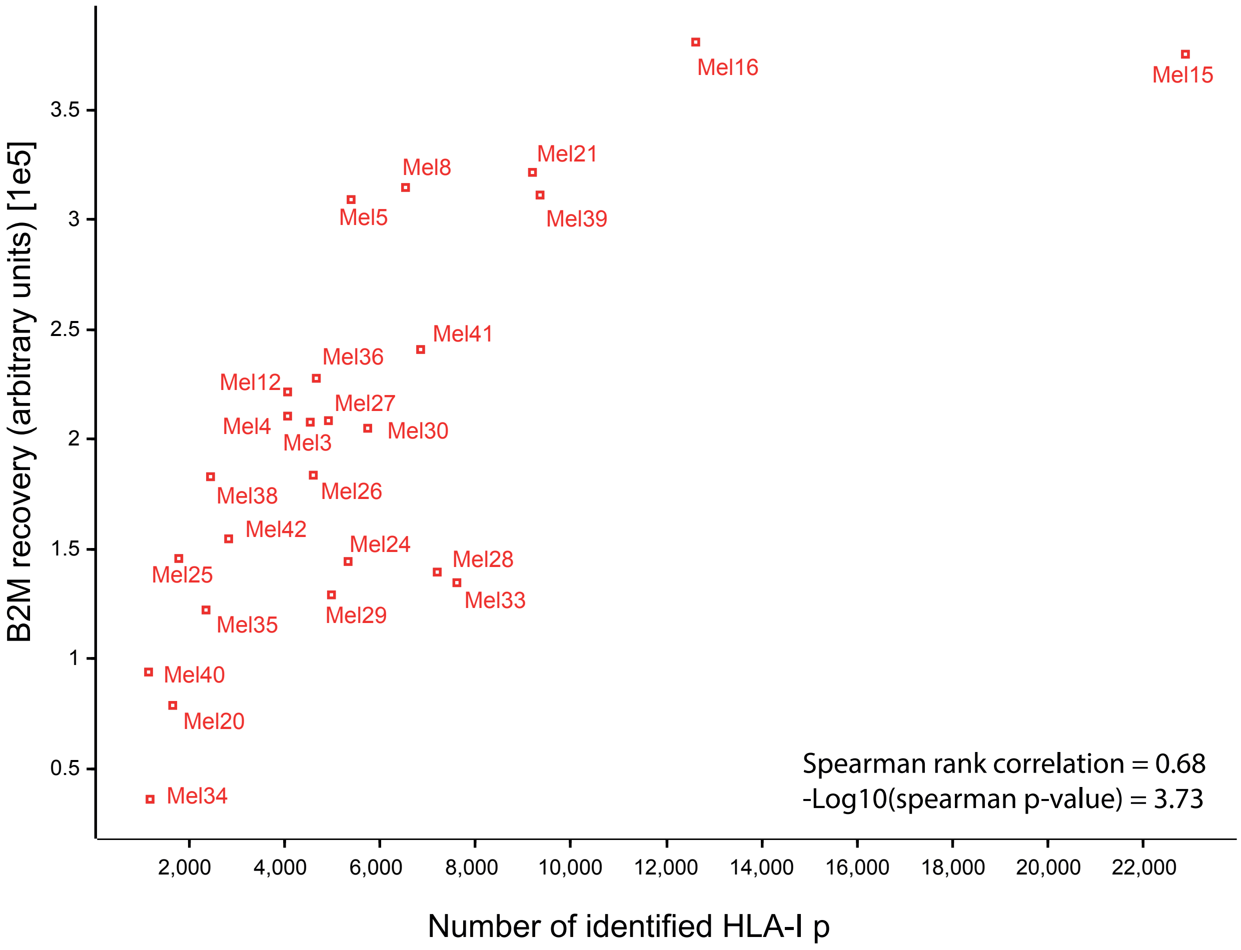

b

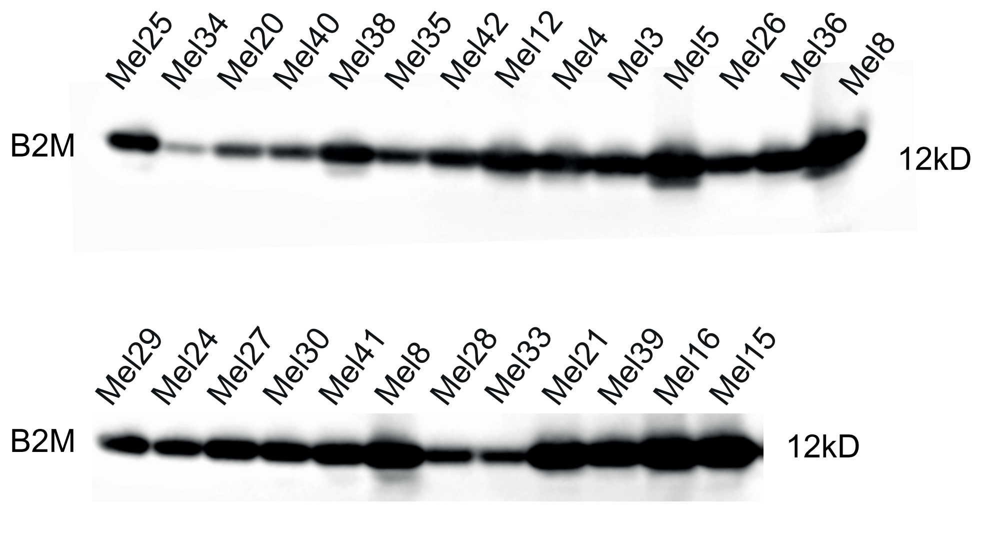

Supplementary Figure 1: Estimation of recovery of HLA complexes after affinity purification. Significant positive correlation between the number of identified peptides in the HLA class I peptidome and amount of the recovered beta-2 microglobulin (B2M) in each tissue sample **(a).** Western Blot results used for quantification of recovered B2M **(b).**

a

b

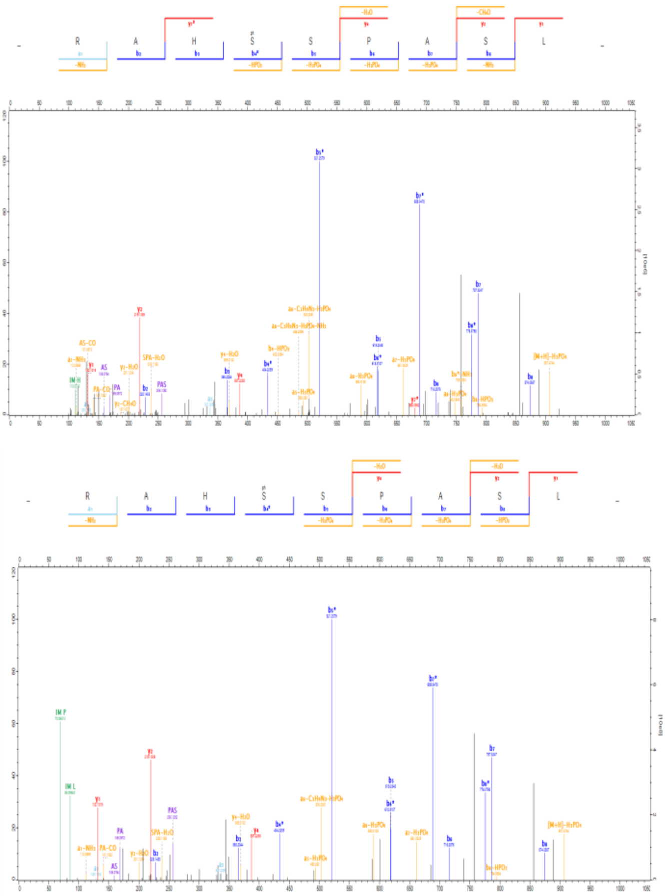

YAP1_RAHS(ph)SPASL_

Supplementary Figure 2. MSMS spectra of YAP1_RAHS(ph)SPASL_ peptide. Eluted HLA binding peptide **(a)** and the corresponding synthetic peptide **(b).**

a

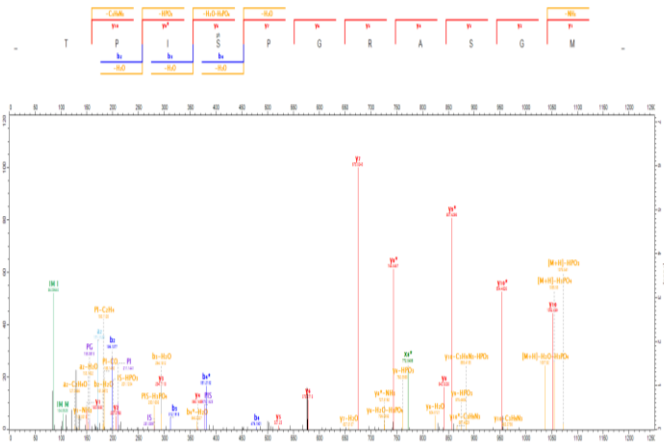

b

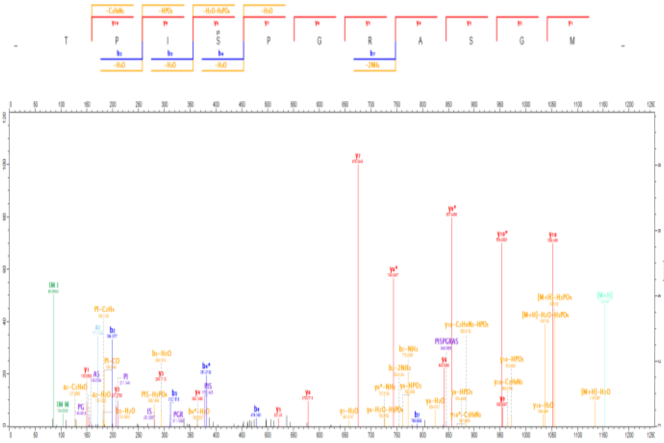

RUNX1_TPIS(ph)PGRASGM_

Supplementary Figure 3. MSMS spectra of RUNX1_TPIS(ph)PGRASGM_ peptide. Eluted HLA binding peptide **(a)** and the corresponding synthetic peptide **(b).**

a

b

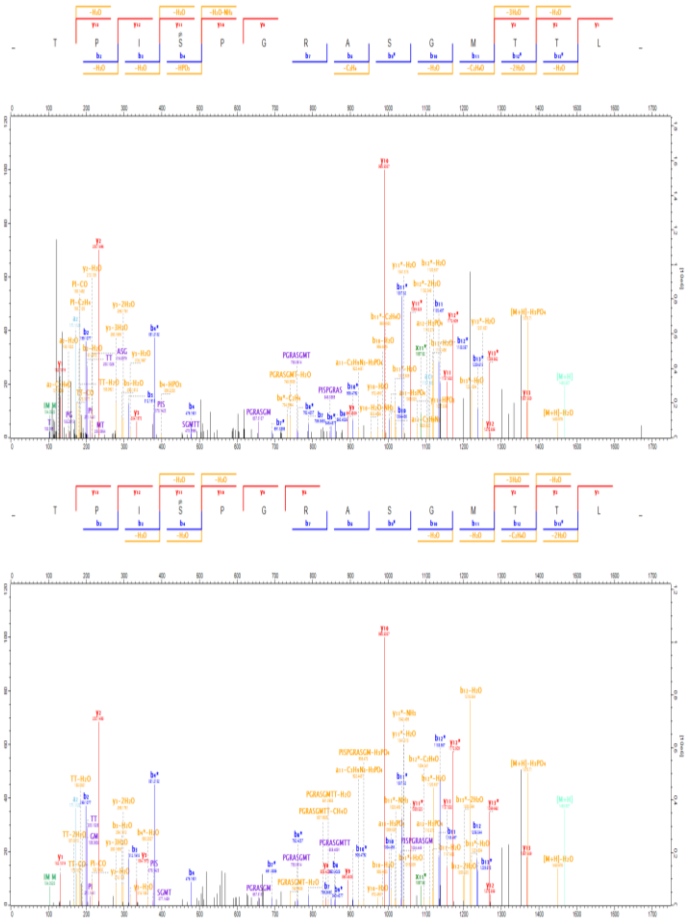

RUNX1_TPIS(ph)PGRASGMTTL_

Supplementary Figure 4. MSMS spectra of RUNX1_TPIS(ph)PGRASGMTTL_ peptide. Eluted HLA binding peptide **(a)** and the corresponding synthetic peptide **(b).**

a

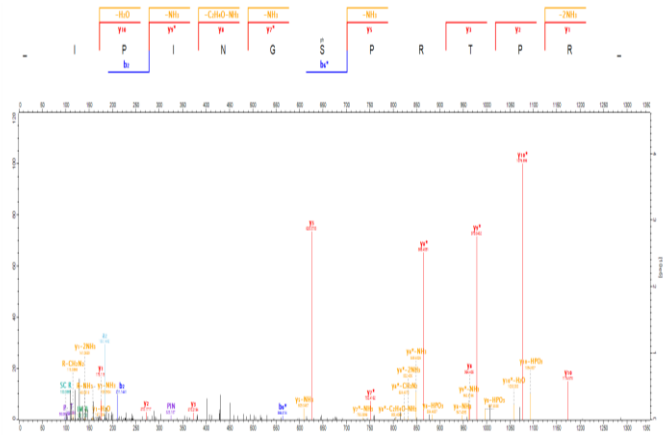

b

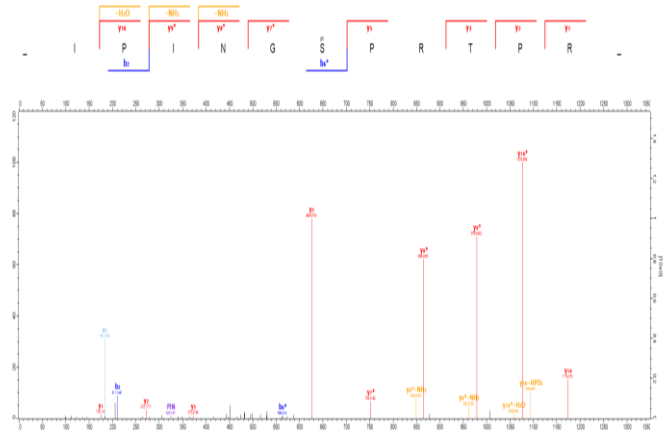

RB1_IPINGS(ph)PRTPR_

Supplementary Figure 5. MSMS spectra of RB1_IPINGS(ph)PRTPR_ peptide. Eluted HLA binding peptide **(a)** and the corresponding synthetic peptide **(b).**

a

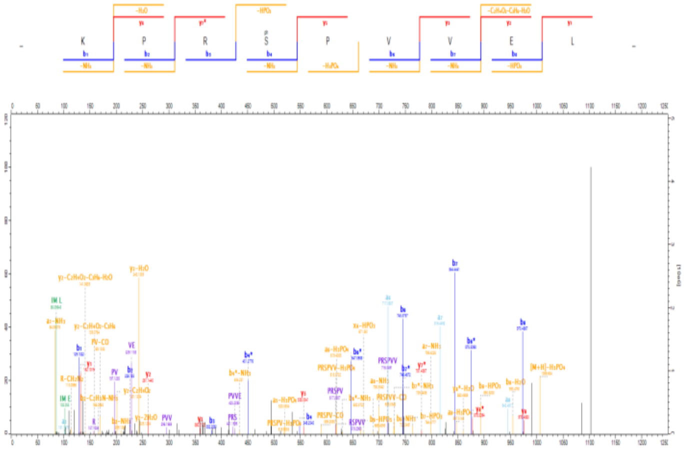

b

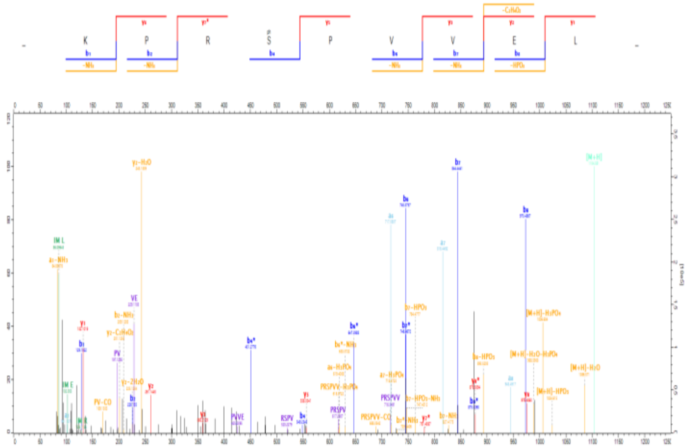

ADRBK1_KPRS(ph)PVVEL_

Supplementary Figure 6. MSMS spectra of ADRBK1_KPRS(ph)PVVEL_ peptide. Eluted HLA binding peptide **(a)** and the corresponding synthetic peptide **(b).**

a

b

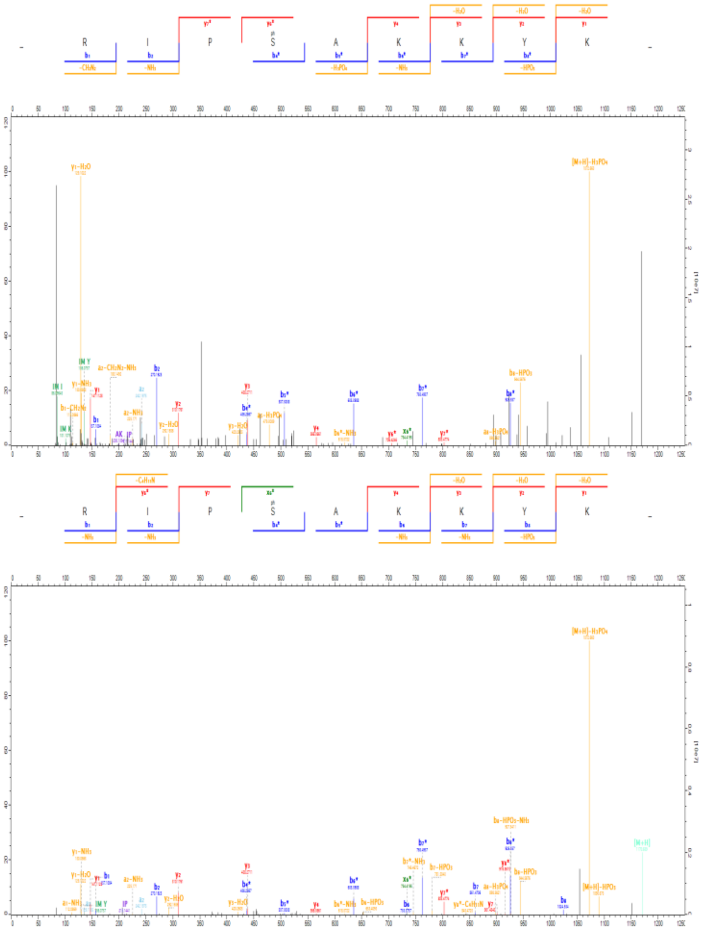

PEA15_RIPS(ph)AKKYK_

Supplementary Figure 7. MSMS spectra of PEA15_RIPS(ph)AKKYK_ peptide. Eluted HLA binding peptide **(a)** and the corresponding synthetic peptide **(b).**

a

b

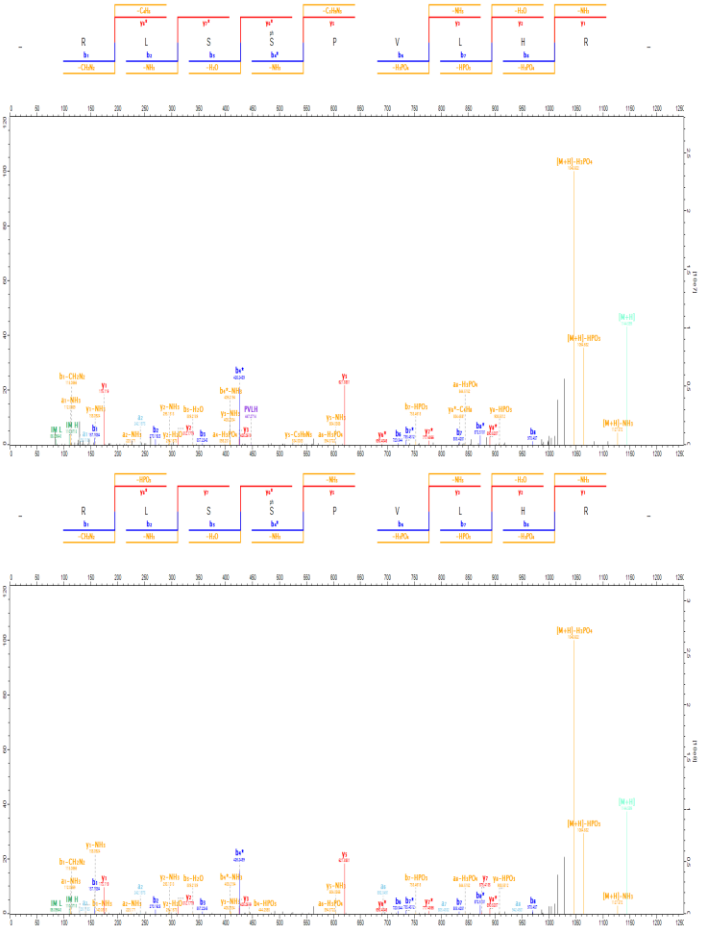

DBN1_RLSS(ph)PVLHR_

Supplementary Figure 8. MSMS spectra of DBN1_RLSS(ph)PVLHR_ peptide. Eluted HLA binding peptide **(a)** and the corresponding synthetic peptide **(b).**

a

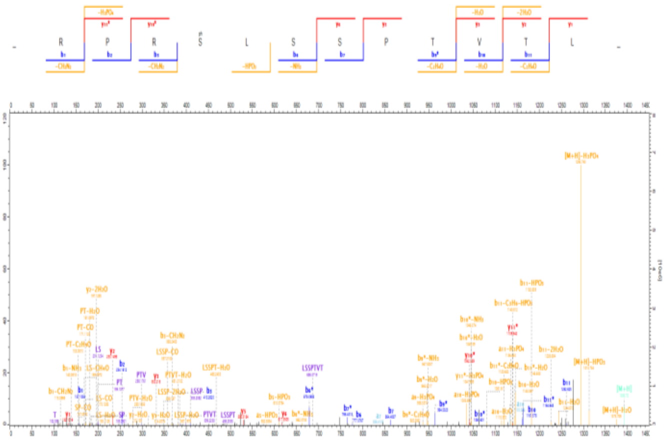

b

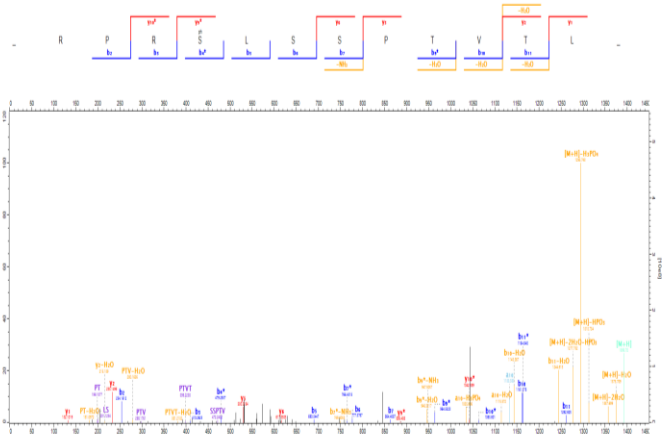

NEDD4L_RPRS(ph)LSSPTVTL_

Supplementary Figure 9. MSMS spectra of NEDD4L_RPRS(ph)LSSPTVTL_ peptide. Eluted HLA binding peptide **(a)** and the corresponding synthetic peptide **(b).**

a

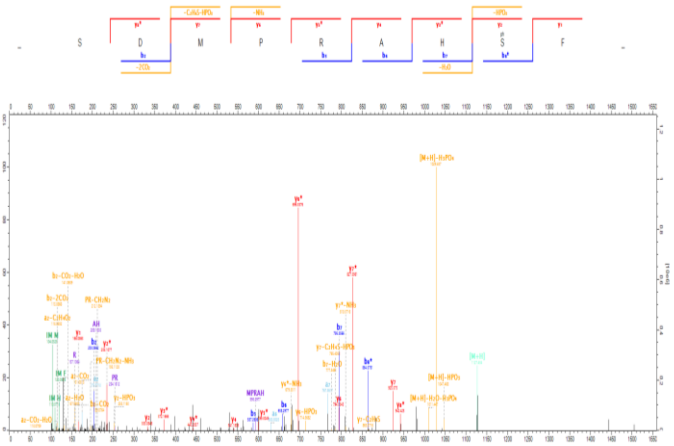

b

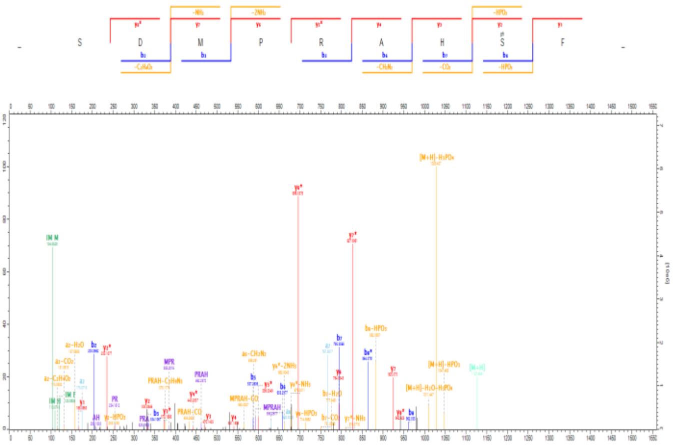

SH3BP2_SDMPRAHS(ph)F_

Supplementary Figure 10. MSMS spectra of SH3BP2_SDMPRAHS(ph)F_ peptide. Eluted HLA binding peptide **(a)** and the corresponding synthetic peptide **(b).**

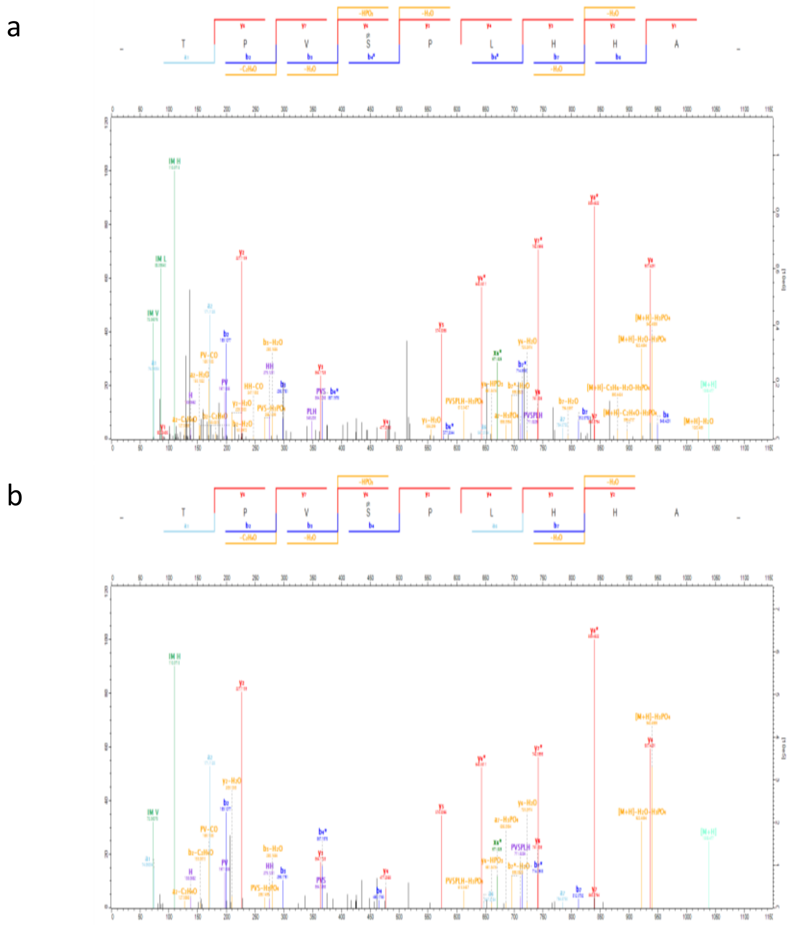

ETV1_TPVS(ph)PLHHA_

Supplementary Figure 11. MSMS spectra of ETV1_TPVS(ph)PLHHA_ peptide. Eluted HLA binding peptide **(a)** and the corresponding synthetic peptide **(b).**

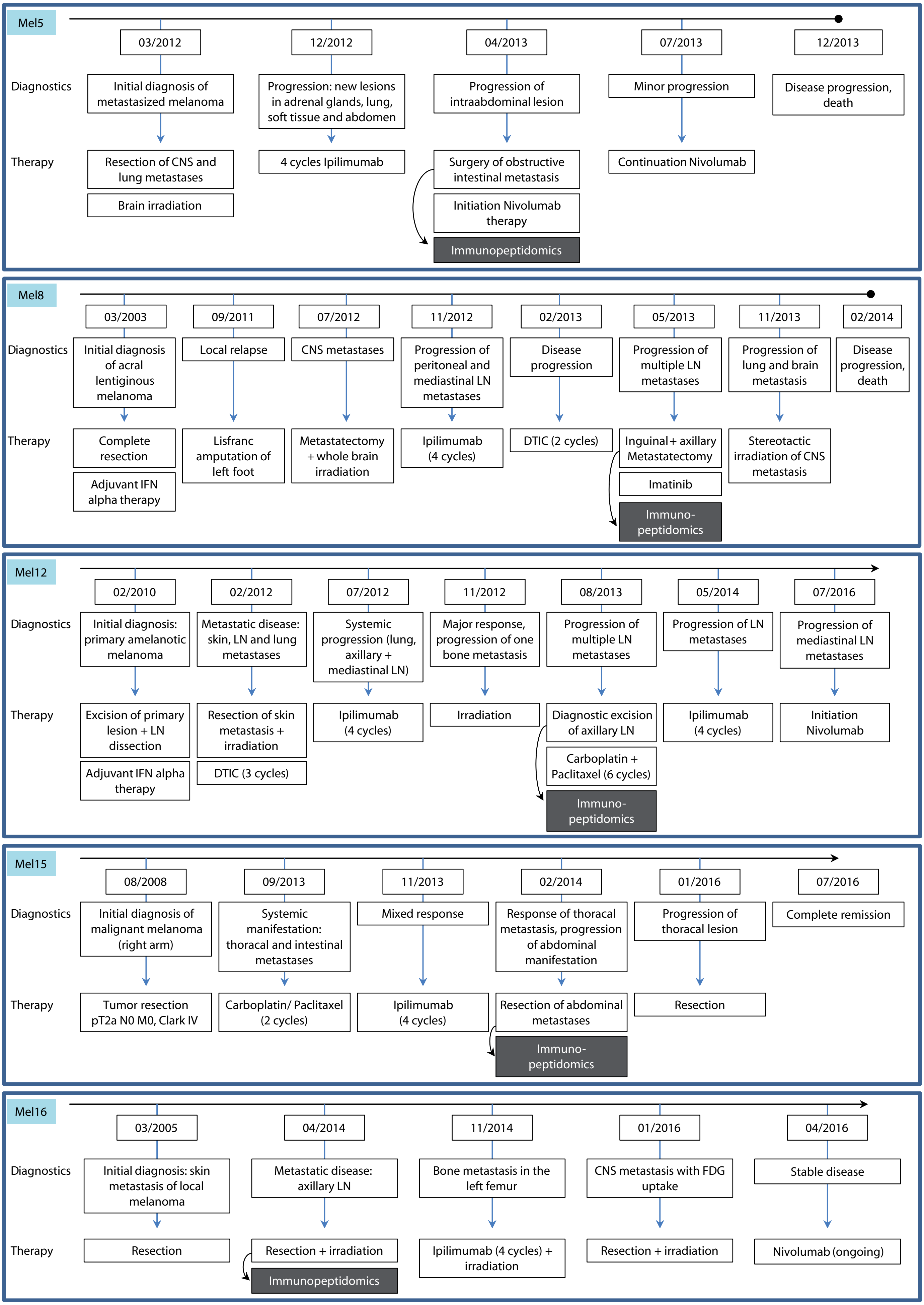

Supplemental Figure 12. Clinical courses of patients Mel5, Mel8, Mel12, Mel15 and Mel16. CNS = central nervous system; LN = lymph node; DTIC = Dacarbazine.

a

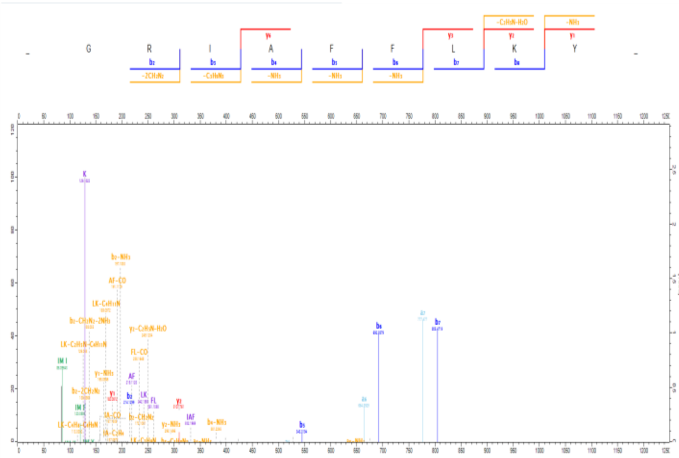

b

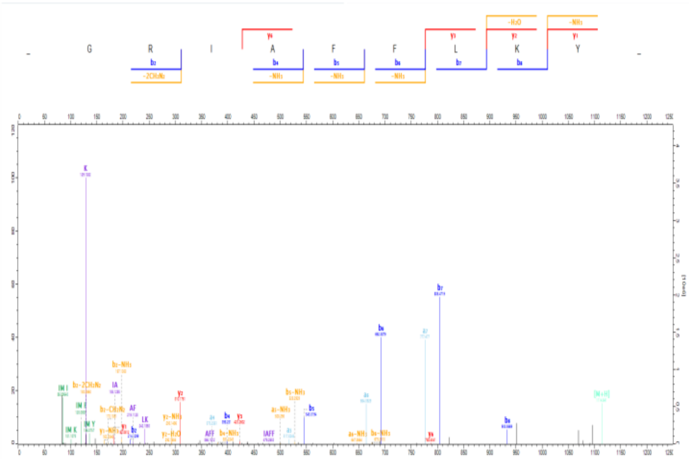

SYTL4_358mut

Supplementary Figure 13. MSMS spectra of SYTL4_358mut peptide. Eluted HLA binding peptide **(a)** and the corresponding synthetic peptide **(b).**

a

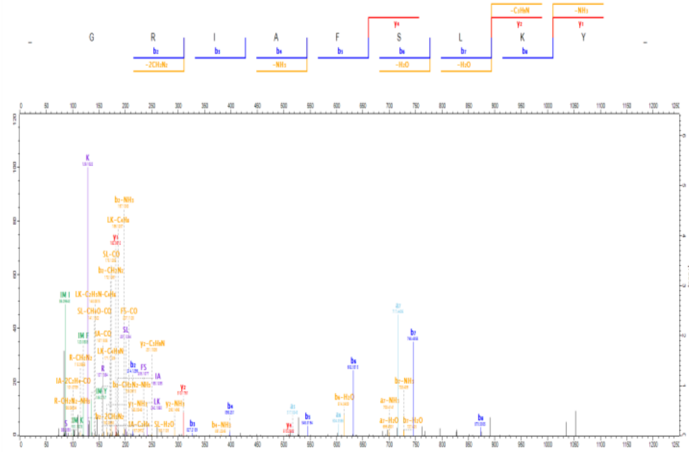

b

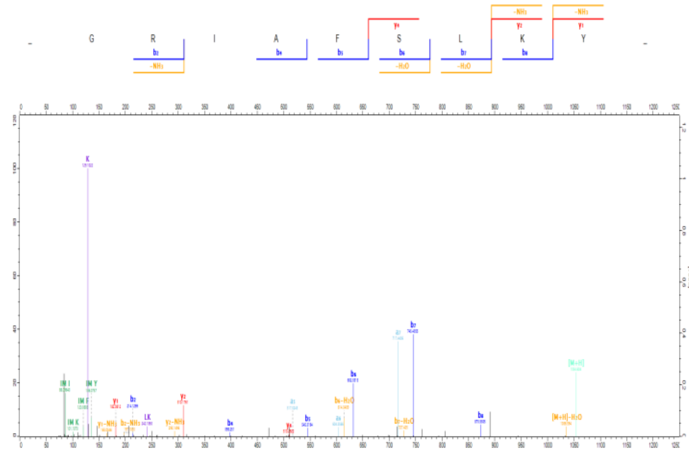

SYTL4_358wt

Supplementary Figure 14. MSMS spectra of SYTL4_358wt peptide. Eluted HLA binding peptide **(a)** and the corresponding synthetic peptide **(b).**

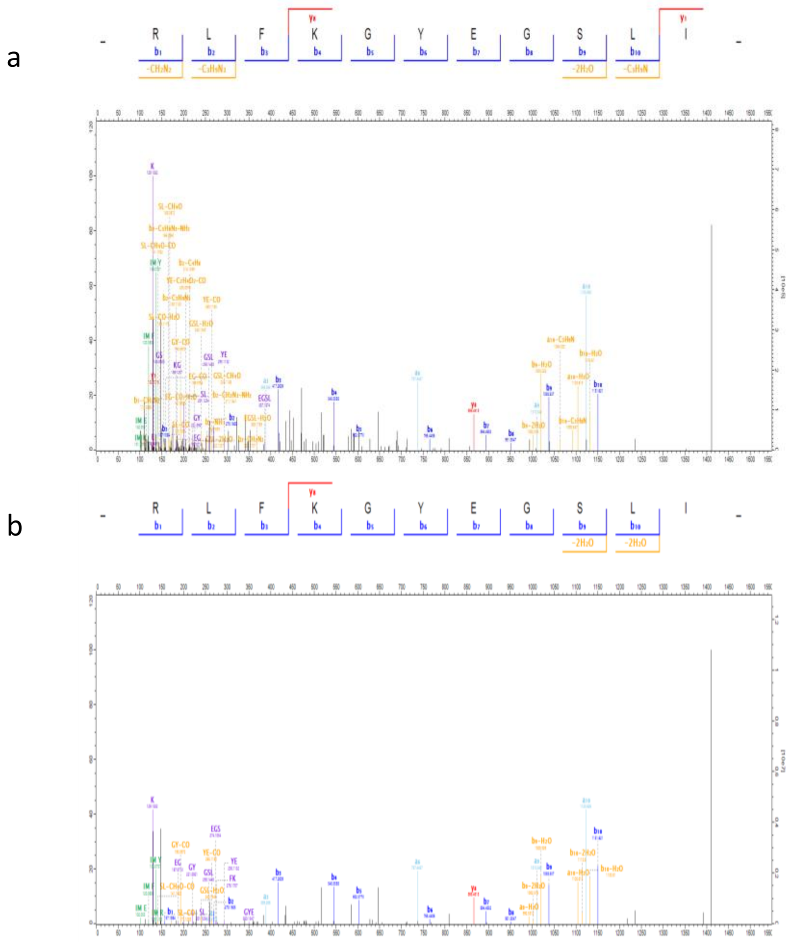

RBPMS_45mut

Supplementary Figure 15. MSMS spectra of RBPMS_45mut peptide. Eluted HLA binding peptide **(a)** and the corresponding synthetic peptide **(b).**

a

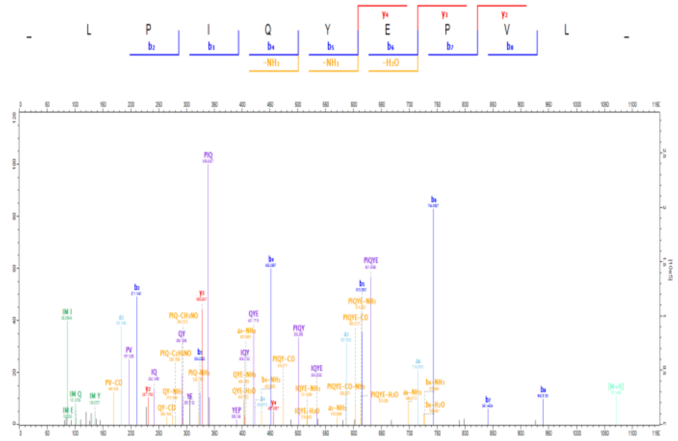

b

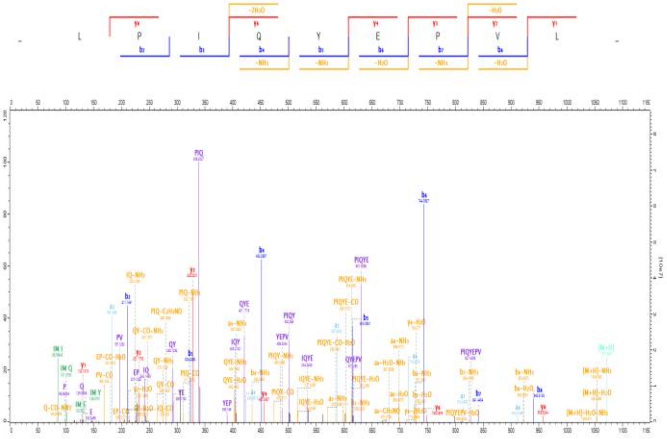

SEC23A_ 169mut

Supplementary Figure 16. MSMS spectra of SEC23A_ 169mut peptide. Eluted HLA binding peptide **(a)** and the corresponding synthetic peptide **(b).**

a

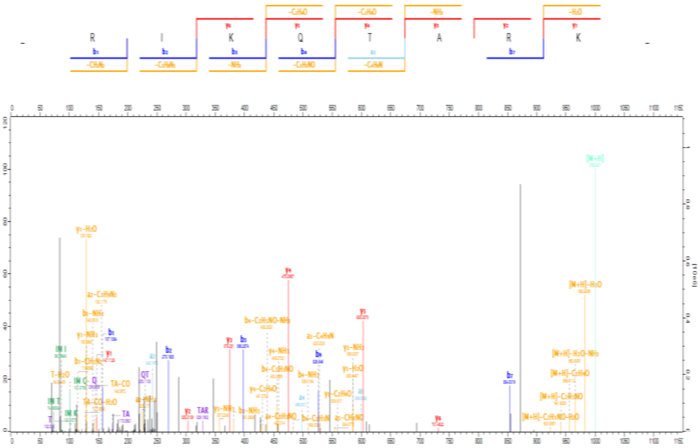

b

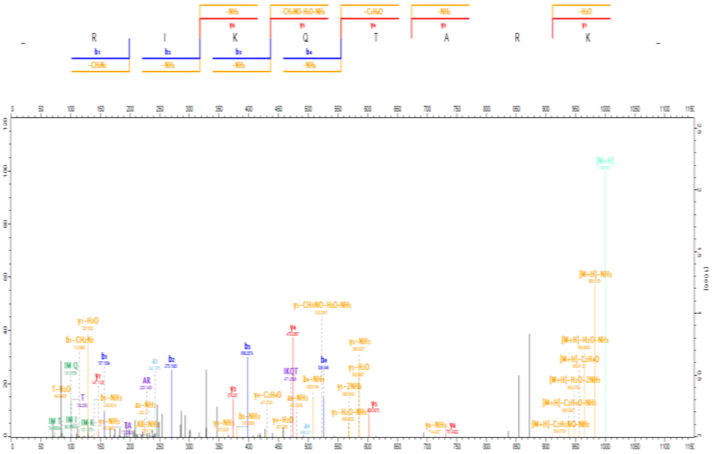

H3F3C_3mut

Supplementary Figure 17. MSMS spectra of H3F3C_3mut peptide. Eluted HLA binding peptide **(a)** and the corresponding synthetic peptide **(b).**

a

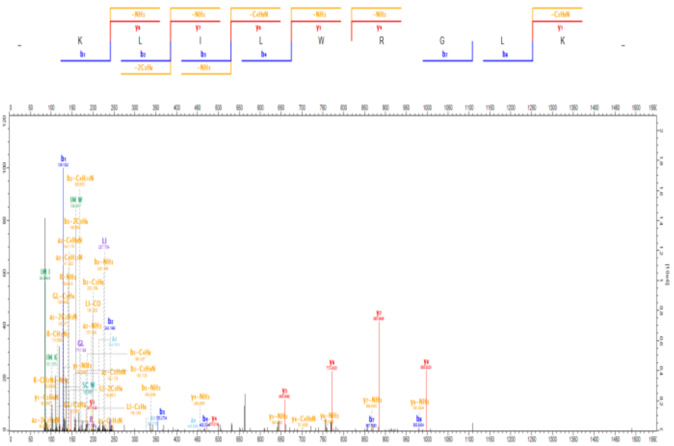

b

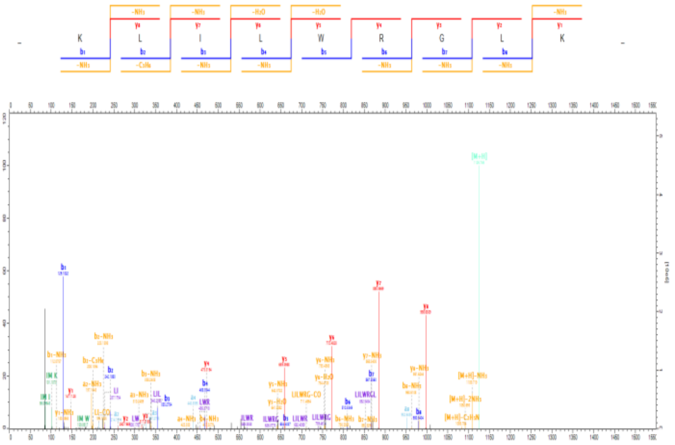

NCAPG2_332mut

Supplementary Figure 18. MSMS spectra of NCAPG2_332mut peptide. Eluted HLA binding peptide **(a)** and the corresponding synthetic peptide **(b).**

a

b

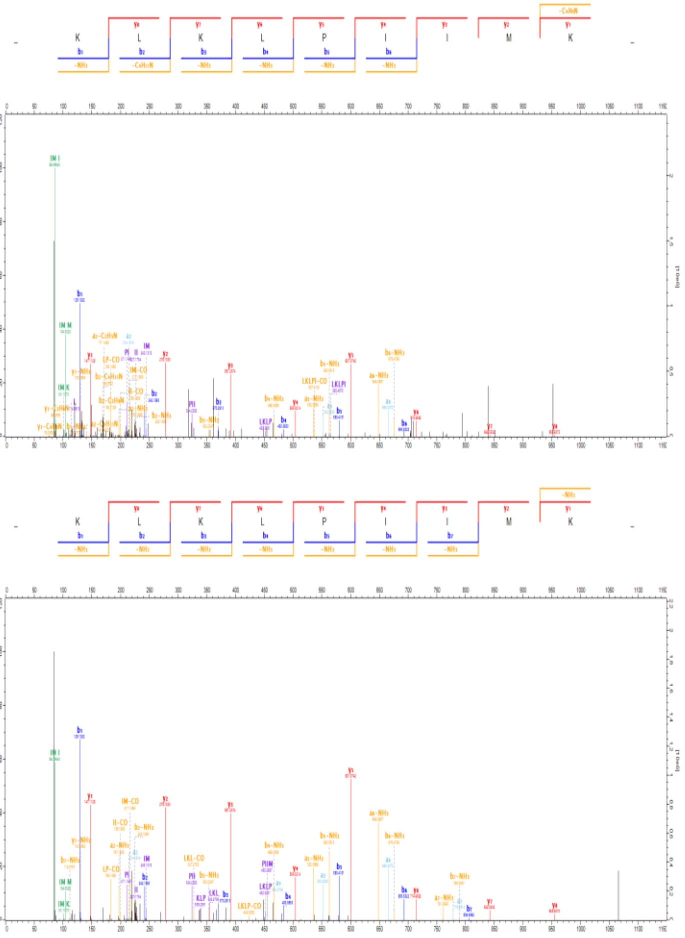

AKAP6_1477mut

Supplementary Figure 19. MSMS spectra of AKAP6_1477mut peptide. Eluted HLA binding peptide **(a)** and the corresponding synthetic peptide **(b).**

a

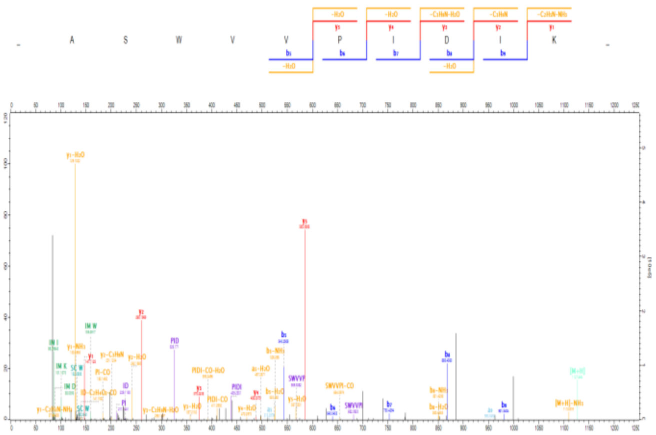

b

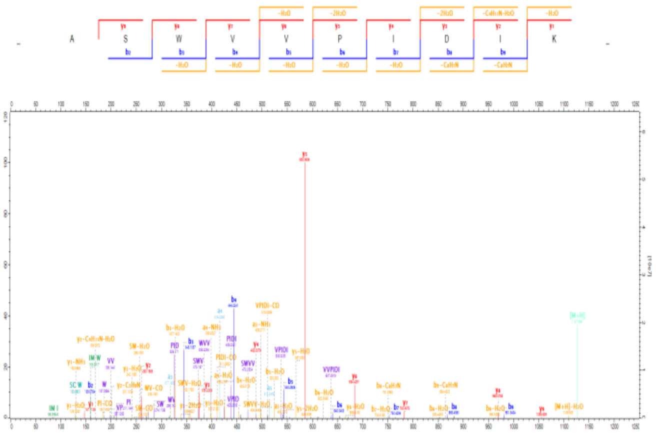

MAPK3K9_679mut

Supplementary Figure 20. MSMS spectra of MAPK3K9_679mut peptide. Eluted HLA binding peptide **(a)** and the corresponding synthetic peptide **(b).**

a

b

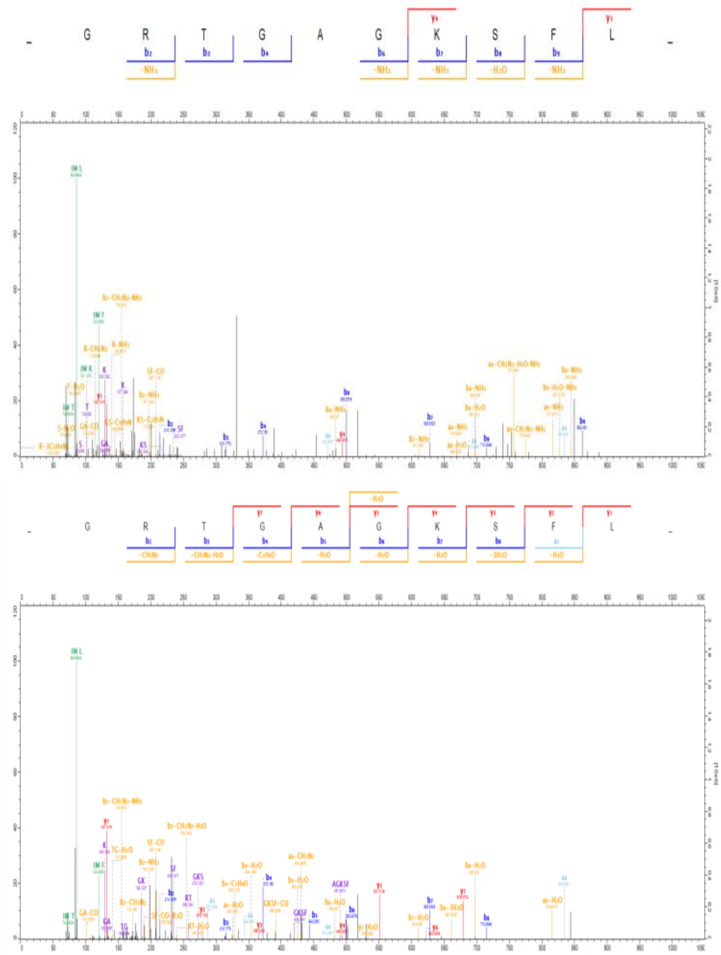

ABCC2_1334mut

Supplementary Figure 21. MSMS spectra of ABCC2_1334mut peptide. Eluted HLA binding peptide **(a)** and the corresponding synthetic peptide **(b).**

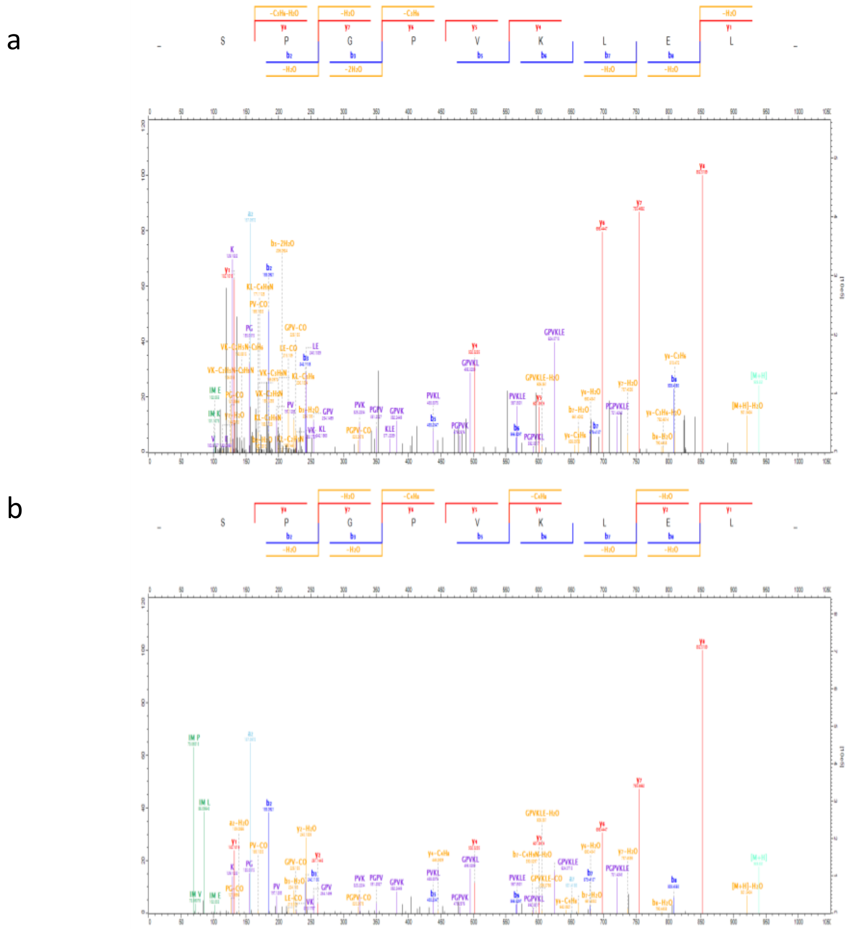

NOP16_169mut

Supplementary Figure 22. MSMS spectra of NOP16_169mut peptide. Eluted HLA binding peptide **(a)** and the corresponding synthetic peptide **(b).**

a

b

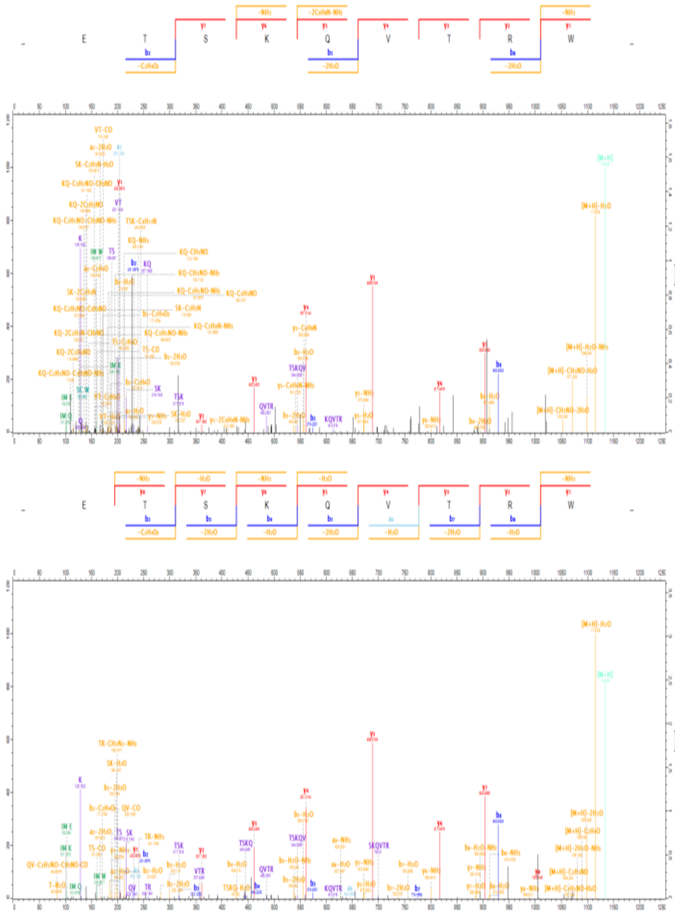

GABPA_158mut

Supplementary Figure 23. MSMS spectra of GABPA_158mut peptide. Eluted HLA binding peptide **(a)** and the corresponding synthetic peptide **(b).**

a

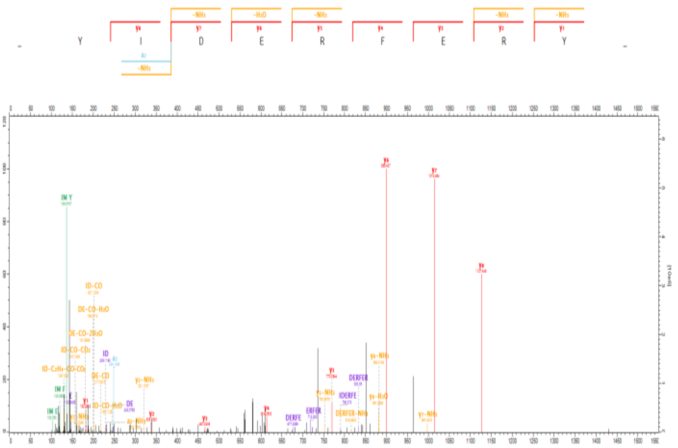

b

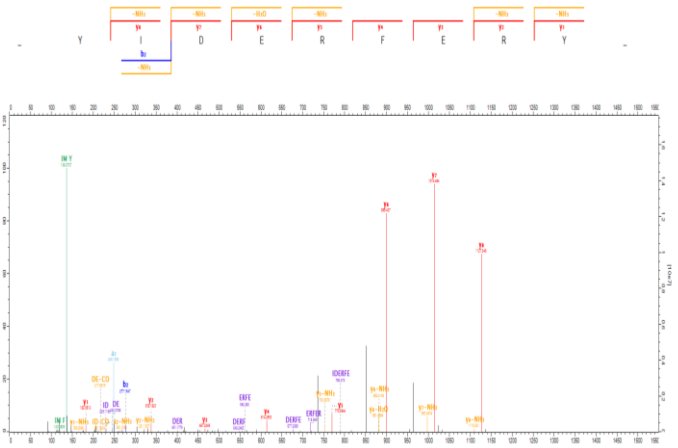

SEPT2_121mut

Supplementary Figure 24. MSMS spectra of SEPT2_121mut peptide. Eluted HLA binding peptide **(a)** and the corresponding synthetic peptide **(b).**

Supplementary Table 1: General patient information

Patient	Gender	Age at biopsy	Location of biopsy
Mel3	female	56	Soft tissue
Mel4	male	80	Bone, soft tissue
Mel5	male	50	Lymph node
Mel8	female	66	Lymph node
Mel12	male	65	Lymph node
Mel15	male	63	Small intestine
Mel16	female	61	Lymph node
Mel20	male	78	Lymph node
Mel21	male	60	Lymph node
Mel24	male	37	Lymph node
Mel25	male	44	Lymph node
Mel26	female	69	Lymph node
Mel27	female	71	Lymph node
Mel28	female	66	Lymph node
Mel29	female	67	Lymph node
Mel30	female	68	Lymph node
Mel33	male	54	Lymph node
Mel34	male	37	Lung
Mel35	male	43	Lymph node
Mel36	male	40	Lymph node
Mel38	male	59	Adrenal gland
Mel39	male	72	Lymph node
Mel40	male	91	Soft tissue
Mel41	male	68	Lymph node
Mel42	female	69	Soft tissue

Supplementary Table 2: Information of patients selected for neoepitope identification

Patient	Stage [A]	Stage at biopsy	LDH [B]	S100 [C]	Defined mutations	HLA type [D]
Mel5	IV	M1C	426	1253	-	A*01, A*25, B*08, B*18
Mel8	IV	M1C	340	668	cKIT p.L576P (Exon 11)	A*01:01:01, A*03:01:01, B*07:02:01, B*08:01:01, C*07:01:01, C*07:02:01
Mel12	IV	M1C	266	56	-	A* 01:01:01, B* 08:01:01, C* 07:01:01
Mel15	IV	M1C	174	82	-	A*03:01:01, A*68:01:01, B*27:05:02, B*35:03:01, C*02:02:02, C*04:01:01
Mel16	IV	M1C	215	94	BRAF V600E	A*01:01:01, A*24:02:01, B*07:02:01, B*08:01:01, C*07:01:01, C*07:02:01

[A] Tumor staging according to Union international contre le cancer (UICC)

[B] At time of metastasectomy (U/L), physiological range < 244 U/L

[C] At time of metastasectomy (pg/ml), physiological range < 100pg/ml

[D] Mel5: determined by HLA miner; all other patients: typed by next-generation sequencing

Supplementary Table 3: MS data of mutated peptide ligands and their synthetic counterparts

					HLA peptide			Syntetic peptide		
Gene Name	Sequence (Position)	ENSEMBL Transcript ID	a.a alteration	Confidence	Score- Peptide spectrum match (PSM) andromeda score (arbitrary units)	Delta score - Score difference between the highest scoring PSM and the next best scoring PSM with a different aa sequence	Retenion time (min)	Score- Peptide spectrum match (PSM) andromeda score (arbitrary units)	Delta score - Score difference between the highest scoring PSM and the next best scoring PSM with a different aa sequence	Retenion time (min)
SYTL4	GRIAFFLKY (358-366)	ENSP00000263033	S363F	High	113	77	83	116	76	87
SYTL4	GRIAFSLKY (358-366)	ENSP00000263033	None	Not assigned	84	44	68	84	42	64
RBPMS	RLFKGYEGSLIK (45-56)	ENSP00000428675	P46L	High	110	62	55	146	88	59
SEC23A	LPIQYEPVL (52-60)	ENSP00000306881	P52L	High	107	58	97	166	70	106
H3F3C	RIKQTARK (3-10)	ENSP00000339835	T4I	High	101	37	6	118	53	14
NCAPG2	KLILWRGLK (332-340)	ENSP00000387007	P333L	High	93	19	66	195	195	68
AKAP6	KLKLPIIMK (1477-1485)	ENSP00000280979	M1482I	High	87	70	66	138	85	68
MAPK3K9	ASWVVPIDIK (680-689)	ENSP00000451263	E689K	Low	76	3	82	182	77	99
ABCC2	GRTGAGKSFL (1334-1343)	ENSP00000359478	S1342F	Low	72	4	37	105	33	34
NOP16	SPGPVKLEL (161-169)	ENSP00000479445	P169L	High	76	32	65	120	59	71
GABPA	ETSKQVTRW (158-166)	ENSP00000346886	E161K	High	83	40	29	167	75	31
SEPT2	YIDERFERY (121-129)	ENSP00000375834	Q125R	High	52	9	49	116	52	54
